# The pharmaceutical effect of Korean wild ginseng residue extract on the performance, microbiota quality, cytokine expression, and the ginseng saponin content of laying hen

**DOI:** 10.1016/j.psj.2024.103467

**Published:** 2024-01-14

**Authors:** Habeeb Tajudeen, SangHun Ha, Abdolreza Hosseindoust, JunYoung Mun, Serin Park, Choi Pok Su, Elick Kinara, JinSoo Kim

**Affiliations:** Department of Animal Industry Convergence, Kangwon National University, Chuncheon, 24341, Republic of Korea

**Keywords:** egg production, microbiota, performance, saponin

## Abstract

In this study, a total of 312 Hyline brown laying hen of 1.92 ± 0.12 kg acquired at 24-wk old were employed to evaluate the pharmaceutical effect of Korean wild ginseng residue extract administered via drinking water on the performance, microbiota quality, cytokine expression, and the ginsenoside saponin (**GS**) content of laying hen for 12 wk. In the experiments, basic feed (**CON**) was compared with basic feed + 0.05% wild ginseng in drinking water (WGD1), basic feed + 0.1% wild ginseng in drinking water (**WGD2**), and basic feed + 0.5% wild ginseng in drinking water (**WGD3**). At the end of study, hen-day egg production (**HDEP**), average egg weight (**AEW**), and egg mass (**EM**) were linearly higher (*p* < 0.05) in WGD3 at wk 30 to 33, 34 to 37 wk, and the cumulative wk compared with CON. Feed conversion ratio (**FCR**) was linearly lower in WGD3 at 34 to 37 wk, and the cumulative wk compared with CON. Relative expression of tumor necrosis factor alpha (**TNF-α**) was linearly lower (*p* < 0.05) in the WGD3 at wk 30 to 33, and 34 to 37 wk compared with CON. The GS in egg yolk was linearly higher (*p* < 0.05) in laying hens supplemented the WGD3 treatment at wk 34 to 37, while the fecal microflora quantity of *Lactobacillus* was linearly higher (*p* < 0.05) in WGD3 at wk 30 to 33, till 34 to 37 wk, and *Escherichia* coli (*E. coli*) was linearly lower (*p* < 0.05) in the WGD2 and WGD3 from 2637 wk compared with CON. We concluded the result in HDEP, AEW, EM, and FCR were due to the increase in GS content, tentatively leading to an improvement in the TNF-α, and fecal microflora quality such as *Lactobacillus* and *E. coli* in the WGD3. We therefore recommend the use of WGD3 at application level 0.5% in drinking water for optimum laying performance from 30 to 37 wk.

## INTRODUCTION

One of the numerous reasons responsible for the expeditious growth of the poultry industry is the rapid production cycle and its contribution to food security which provides significant means of obtainable nutrients to humans via its meat and egg products (Grzinic et al., 2023). However, the industry still encounters several hurdles due to the continuous increase in the human population (Alghirani et al., 2022). In an attempt to meet the rising demand for chicken meat and egg production, interminable studies such as the use of growth stimulants and antibiotics are being carried out to improve the health and productivity of layer hens (Agustono et al., 2023). However, the recent depleting value for antibiotics as growth enhancers and bactericides in poultry has instigated the consideration of other alternatives (Tajudeen et al., 2022).

Phytogenic such as wild ginseng (**WG**) contains bioactive compounds which include ginseng saponin (**GS**), lignans, peptides, polyacetylenes, and alkaloids, making it a potent source of traditional medications for centuries in the Eastern part of Asia due to its multifaceted immune and energy boosting ability with anti-oxidative and anti-inflammatory capacity (Yan et al., 2011; Peng et al., 2023). This antioxidant and anti-inflammatory activity is vital for cellular health and may contribute to ginseng's reported anti-aging effects, and has been proven to improve performance (Su et al., 2023; Tajudeen et al., 2023). Thus, serving as a viable alternative to antibiotics. However, the growth of WG is naturally slow with a longer adaptation period and exposure to various environmental conditions (Farley, 2023). This has been speculated to improve its potency compared to the cultivated ginsengs, resulting in its expensive nature and cost (Yan et al., 2010; Farley, 2023).

Ginseng root residue which is the by-product obtained from processing ginseng beverages is a viable and cheaper alternative, with reasonable amount of the saponin active ingredients including GS yet to be completely explored (Hsu et al., 2022; Li et al., 2023). Our current research suggested the method could be an efficient alternative to the mass production of cheaper WG for improving laying performance in hens. The administration of herbs in liquid form has also been reported to be one of the easiest means to facilitate its absorption in the digestive stream (Porqueddu, 2017) However, there is a limited report on the supplementation of water-based WG root residue to laying hen via drinking water (Sharma et al., 2020; Mutlu and Yildirim, 2020).

Therefore, the objective of this experiment was to investigate the pharmaceutical effect of water-based WG root residue supplementation on the laying performance, GS content, cytokine expression, and the microflora quantum of laying hen.

## MATERIALS AND METHODS

### Ethical Approval

The laying hen experiment was conducted at the Kangwon National University's laying hen facility after receiving official approval from the Animal Experimental Ethics Committee with an ethical code (KW-220413-1).

### Experimental Animals, Feed and Housing

A total of 312 Hyline brown laying hen of 1.92 ± 0.12 kg acquired at 24-wk old were subjected to a completely randomized design of 4 treatments and 6 repetitions with 13 birds per replicate. The first 14 d of the experiment was to allow the birds an adaptation period, after which they were subjected to the experimental drinking water for 12 wk (wk 26–29 as phase 1, wk 30–33 as phase 2, and wk 34–37 as phase 3). We divided the experiment into 4 groups as in basic feed (**CON**), **WGD1** (basic feed + 0.05% wild ginseng in drinking water), **WGD2** (basic feed + 0.1% wild ginseng in drinking water), and **WGD3** (basic feed + 0.5% wild ginseng in drinking). In this study, economic reasons were taken into consideration when determining the inclusion level at lower doses. Ginseng has been evaluated between 1.0 and 5% by previous researchers (Yan et al., 2011; Hou et al., 2013). We, therefore evaluated the efficacy using minute doses via drinking water but with by-products from WG raised for 15 years as it is generally recognized that the potency of WG is proportional to the duration, specie, and the parts incorporated. (Hou et al., 2013). During the trial period, the laying hens were kept in a 3-dimensional pen with excellent ventilation of 22 to 24°C temperature and lightening of 16 h per day using clean 7 W LED bulb (Overdrive, L10NA19DIM 3000 K; White). The pens were enriched according to the European union standards for the protection of laying hens (Directive EU; CEC, 1999); each pen was provided 2 periodically tested nipple drinker (Big Dutchman AG, Vechta-Calveslage, Germany) and a feed trough (12 cm), a perch (15 cm long; providing 15 cm/hen), a nest, and a claw-shortening device. Each pen had a total area of 6,400 cm^2^ (providing 1,225 cm^2^/hen) with feeds and water supplied ad-libitum*.* The dietary requirements of the experiment were calculated to meet or surpass the nutrient need as specified by the Hyline brown commercial management guide as stated in Table 1 supplied in mash form**.** The cleaning and other management practices were carried out twice a day (9:30 am and 4:30 pm) throughout the experimental period.

**Table 1:** Experimental feed mixing ratio (air dry basis).

Item	Basic feed
Raw material feed mixing ratio, %	
Corn	62.20
Rice bran	1.53
Soybean meal, crude protein 45%	24.00
Animal fat	1.50
Limestone	8.55
Tricalcium phosphate	1.40
Vitamin-mineral additive	0.32
Sodium chloride	0.31
DL -Methionine	0.19
Sum	100.00
Calculated and determined analysis (%)	
Metabolizable energy, kcal/kg	2,750
Crude protein	16.00
Ash	9.95
Crude fiber	4.11
Crude fat	3.90
Calcium	3.50
Total P	0.48
Available P	0.32
Lysine	0.84
Methionine	0.41
Methionine + cysteine	0.66
Thr	0.61
Trp	0.19

Content in kg: vitamin A, 10,000 IU (retinyl acetate); vitamin D_3_, 2,000 IU (cholecalciferol); vitamin E, 0.25 IU (tocopheryl acetate); vitamin K_3_, 2 mg (menadione dimethylpyrimidine); Vitamin B12, 10 mg (cobalamin); Choline, 250 mg; folic acid, 1 mg; niacin, 30 mg; pantothenic acid, 10 mg; pyridoxine, 3 mg; riboflavin, 6 mg; thiamine, 2 mg; ethoxyquin, 125 mg; cobalt,0.3 mg; copper, 10 mg; iron, 60 mg; iodine, 0.5 mg; manganese, 40 mg; selenium, 0.2 mg; Zinc, 50 mg.

### Preparation of the Water-Based Korean Wild Ginseng Residue Extract

The liquid ginseng from wild roots residue was obtained using the mini multi-functional herb extractor vacuum concentrator with model item TD100-1 (Wenzhou Yinuo, China). This was achieved by adding WG roots residue (a by-product obtained from processing ginseng beverage), water, and ethanol into an extraction tank and subjected to 4 repeated extraction process at 70°C for 10 h. The extract was filtered under pressure using the 70 mm 4-layer 250 mesh filter and then conveyed into a reduced pressure vacuum concentrator where it was concentrated at 500 to 700 mm Hg at 70°C to further activate the active ingredients in WG with its chemical constituents as shown in Table 2.

**Table 2:** Analyzed chemical constituents of ginseng root residue in %.

Ether extract	0.95
Crude fiber	16.95
Crude protein	13.28
Ash	3.51
Total ginsenoside /ginseng saponin	5.23

### Laying Performance and Egg Quality

The hen day egg production (**HDEP**) was calculated using the total number of eggs produced during the period/total number of hens alive in the same period × 100. The quality inspection of eggs including Haugh units (**HU**), yolk and albumin weights, yolk and albumin percentages, and average egg weight (**AEW**) was conducted after each phase using the egg multi-tester (Tohoku Rhythm Co. Tokyo, Japan), and egg mass (**EM**) was calculated via percent HDEP × AEW. The feed conversion ratio (**FCR**) was calculated by considering average daily feed intake (**ADFI**) and AEW. All layer hens were weighed at the conclusion of each phase.

### Fecal Microflora Evaluation

Clean fecal samples from 10 birds per treatments were obtained through the gentle palpation of the cloaca into the collection tube and immediately stored at -80°C until analysis at the conclusion of each phase. Deoxyribonucleic acid (**DNA**) extraction was then performed using the QIAampfast DNA stool small kit Germany, cat. no. 51604 2016. The real-time PCR (quantitative real-time polymerase chain reaction) for the quantifying fecal microbiota such as *Lactobacillus spp., Bifidobacterium spp., Clostridium spp., and Escherichia* coli (***E. coli****)* was then carried out using the method by Tajudeen et al. (2023) with Qiagen 2plex program, Serial Number 0312272, Corbett Research (Corbett Life Science Qiagen, Sydney, New South Wales, Australia). SsoAdvanced universal SYBR GreenSupermix, 2.5 ng/*μ*L forward and reverse primers and 10 ng of DNA were included. The activation of the enzyme was achieved at 95°C, following a 40 cycle of melting at 95°C for 15 s; annealing for the stipulated duration and temperatures according to each primer as shown in Table 3 with beta-actin (**β-actin**) as the housekeeping gene. SYBR green fluorescence signals were captured at 72°C, and known bacterial species were serially diluted 10-fold for standard curve construction before being used for generating the PCR outcomes. To ensure its specificity, the melting curve analysis step was programmed and carried out during the final cycle of each amplification (Stringer et al., 2008; Muhammad et al., 2021).

**Table 3:** Cycling details of primers used for fecal microflora DNA and TNF-α in this study.

Microflora	Primer sequence	Anneal temperature	Cycles

*Lactobacillus* spp.	F: DNA-AGC AGT AGG GAA TCT TCC A R: DNA-CAC CGC TAC ACA TGG AG	54.0°C 53.6°C	40
*Bifidobacterium* spp.	F: DNA-TCG CGT CYG GTG TGA AAG R: DNA-CCA CAT CCA GCR TCC AC	59.4°C 55.9°C	
*Clostridium* spp.	F: DNA-GGC GGC YTR CTG GGC TTT R: DNA-CCA GGT GGA TWA CTT ATT GTG TTA A	62.1°C 56.1°C	
*E. coli* spp.	F: DNA-AAA ACG GCA AGA AAA AGC AG R: DNA-GCG TGG TTA CAG TCT TGC G	55.0°C 58.6°C	
β. Actin	F: DNA-CTC CTT CCT GGG CAT GGA R: DNA-CGC ACT TCA TGA TCG AGT TGA	57.3°C 57.8°C	
Cytokine			
TNF-a NM_204267	F: DNA-GCC CCT GTA ACC AGA TG R: DNA-ACA CGA CAG CCA AGT CAA CG	57°C 60.2°C	
GAPDH NM_204305	F: DNA-AGA ACA TCA TCC CAG CGT CC R: DNA-CGG CAG GTC AGG TCA ACA AC	58.8°C 60.6°C	

### Blood Immune Substance and Gene Extraction Analysis for Cytokine Expression

#### Blood Withdrawal and Blood Isolation

Blood samples of at least 5 mL were slowly collected via the median underwing of 10 birds per treatments using a disposable Luer-Lok syringe and 21-gauge precision glide needle (Becton Dickinson & Co., Franklin Lakes, NJ) and placed in an ice-filled K2EDTA heparin tube (Becton Dickinson Vacutainer, Systems Europe, Meylan, France). The Union 55R refrigerated multifunctional centrifuge, made by Hanil Science Industrial Co., Ltd., Seoul, Republic of Korea was used to centrifuge blood samples for 40 min at 4,000 rpm at 20°C in order to separate peripheral blood mononuclear cells (**PBMCs**). The breakpoint was set at 0, and the final result was separated and kept at −4°C. The messenger ribonucleic acid (**mRNA**) was first extracted from the PBMC using the RNeasy Mini kit from Qiagen, Germany. An Ultraturrax homogenizer (Polytron PT 16,00E, Kinematica, Luzern, Switzerland) was used to homogenize the samples while the cells were trapped in 600 µl of lysis-buffer containing β-mercaptoethanol and RLT-buffer before they were blended and used for further analysis.

#### Reverse Transcription Polymerase Reaction and Quantitative Polymerase Chain Reaction Analysis

To reverse transcribe the mRNA, 250 ng of extracted mRNA and 100 μL of TaqMan Reverse Transcription Reagents (Life Technologies, Carlsbad, CA) were employed in conjunction with 6.2 *μ*L of MultiScribe Reverse Transcriptase, 20 μL dNTP, 2 μL RNAse inhibitor, 5 μL of random hexamers, 2 μL oligo-DT, 10 microliters 10 × buffer, and 25 mM MgCl2. Reverse transcription was carried out in a German-made Eppendorf Flexid Nexus Gradient Master Cycler (SN:6332kl132036), with annealing at 25°C for 10 min and enzyme inactivation at 95°C for 5 min. The relative tumour necrosis alpha (**TNF-α**) was then quantified with 5 *μ*L cDNA, 1.75 μL Aqua dest., 2 μL of TaqMan Master Mix and 0.25 μL of both forward and reverse primers, and glyceraldehyde-3-phosphate dehydrogenase (**GAPDH**) was the housekeeping gene (Bioneer, Daejeon, Korea) Table 3. The qPCR Rotor-Gene Qiagen SN 0312272 (Corbett Research) with cycling temperature of 95°C followed by cycles of melting at 95°C for 15 s; annealing for the periods and temperatures in accordance with each primer; and extension at 72°C at 40 cycles were considered. (Tajudeen et al., 2023)

#### Ginseng Saponin / Ginsenosides in Egg Yolk

The GS content was measured using the methanol extraction method on 12 eggs per treatment. In detail, 2 g of dry matter sample was extracted into 20 mL methanol at a constant water bath temperature of 60°C for 3 h. The solution was extracted under reduced pressure at a reflux condition with temperatures not exceeding 40°C, and the leftover residue was dissolved in 5 mL of distilled water. The dissolved residues were poured into a separatory funnel, and the resulting layers were sorted with 50 mL of chloroform to remove non-polar components such as fat and the organic solvent component. The remaining residue was then rinsed 3 times with 50 mL of ethyl ether. The dissolved GS layer was extracted using saturated n- butanol, and the solvent was withdrawn under reduced pressure at 40°C in an evaporator (IKA RV8, Germany). The total GS content was determined via gravimetric analysis (Hou et al., 2013)

### Statistical Analysis

The data acquired during this study were analyzed statistically adopting one-way ANOVA by the Statistical Analysis System (**SAS** Institute, 2000). Individual pens containing the layer hens per treatment were used as the experimental unit, and variables evaluated multiple times were expressed as repeated measures. The Duncan's multiple range test was employed to ascertain treatment differences, while the effects of WGD levels were tested linearly and quadratically using orthogonal polynomial contrasts. Data were expressed as means and standard with values of p<0.05 were classified as statistically significant.

## RESULTS

### Laying Performance

The effect of supplementing WG in drinking water on the performance of laying hen is presented in Table 4. There was no significant difference in ADFI and BW across all treatments and wk. There was also no significant difference in HDEP, AEW, EM, and FCR in wk 26 to 29, as well as in wk 30 to 33 for FCR. However, HDEP, AEW, and EM were linearly higher (*p* < 0.05) in wk 30–33, wk 34–37, and the cumulative wk at the WGD3 compared with CON. FCR was linearly lower (*p* < 0.05) at WGD3 compared with CON at wk 34–37, and the cumulative wk.

**Table 4:** Effects of water supplemented wild ginseng on laying performance.

Items^1^	CON	WGD1	WGD2	WGD3	SEM	*Linear*	*Quadratic*
ADFI, g/hen/d							
26–29 wk	111.09	112.61	113.31	111.70	2.976	0.993	0.935
30–33 wk	111.71	112.40	113.32	111.40	2.594	0.992	0.490
34–37 wk	113.10	110.91	111.90	110.31	1.879	0.233	0.810
Cumulative (26–37 wk)	112.23	111.96	112.82	111.13	1.788	0.670	0.581
BW, kg							
26–29 wk	1.97	2.01	1.95	1.98	0.077	0.916	0.899
30–33 wk	1.99	2.07	2.00	2.02	0.068	0.938	0.486
34–37 wk	2.02	2.08	2.04	2.06	0.073	0.726	0.634
Cumulative (26–37 wk)	1.99	2.05	1.99	2.01	0.058	0.898	0.601
HDEP, %							
26–29 wk	93.58	93.88	94.24	94.46	0.660	0.170	0.933
30–33 wk	93.16^c^	93.42^ab^	93.86^ab^	94.58^a^	0.535	0.013	0.534
34–37 wk	92.46^c^	92.72^bc^	93.77^ab^	94.28^a^	0.452	<0.001	0.699
Cumulative (26–37 wk)	93.06^b^	93.33^b^	93.95^ab^	94.44^a^	0.351	0.001	0.668
AEW, g							
26–29 wk	58.98	59.23	59.66	59.91	0.703	0.165	0.952
30–33 wk	59.43^ab^	59.86^ab^	60.67^ab^	61.27^a^	0.738	0.015	0.880
34–37 wk	60.61^b^	60.89^ab^	61.72^ab^	62.36^a^	0.605	0.006	0.673
Cumulative (26–37 wk)	59.67^b^	59.99^ab^	60.68^ab^	61.18^a^	0.612	0.016	0.842
Egg mass, g/hen/d							
26–29 wk	55.19	55.60	56.21	56.59	0.660	0.035	0.976
30–33 wk	55.36^b^	55.91^ab^	56.95^ab^	57.95^a^	0.820	0.004	0.696
34–37 wk	56.04^b^	56.45^bc^	57.87^ab^	58.79^a^	0.591	<0.001	0.552
Cumulative (26–37 wk)	55.53^b^	55.98 ^b^	57.01^ab^	57.78^a^	0.576	0.001	0.705
FCR, g feed/g egg							
26–29 wk	2.03	2.03	2.02	1.97	0.052	0.308	0.597
30–33 wk	2.02	2.01	1.99	1.92	0.058	0.118	0.479
34–37 wk	2.02^a^	1.97^ab^	1.93^ab^	1.88^b^	0.045	0.005	0.960
Cumulative (26–37 wk)	2.02^a^	2.00^ab^	1.98^ab^	1.92^b^	0.038	0.022	0.544

SEM, standard error of means; ADFI, average daily feed intake; BW, body weight; HDEP, hen day egg production; AEW, average egg weight; FCR, feed conversion ratio.

1 CON, drinking water without wild ginseng; WGD1, 0.05% wild ginseng in drinking water; WGD2, 0.1% wild ginseng in drinking water; WGD3, 0.5% wild ginseng in drinking water. The same letter superscript indicates no significant difference, whereas different letter superscript indicates significant differences (*P* < 0.05).

### Egg Quality

The effect of supplementing WG in drinking water on the egg quality of laying hen is presented in Table 5. There was no significant difference in HU, yolk weight, albumin weight, yolk percentage, and albumin percentage across all treatments and wk.

**Table 5:** Effects of water supplemented wild ginseng on egg quality.

Items^1^	CON	WGD1	WGD2	WGD3	SEM	*Linear*	*Quadratic*
Haugh units							
26–29 wk	77.60	79 .24	79.08	78.79	2.970	0.720	0.652
30–33 wk	77.43	80.05	78.12	78.33	4.481	0.957	0.708
34–37 wk	77.02	79.72	77.78	77.97	4.449	0.949	0.694
Cumulative (26–37 wk)	77.35	79.67	78.33	78.36	3.755	0.888	0.672
Yolk weight, g							
26–29 wk	16.29	16.31	16.83	16.85	0.283	0.066	0.996
30–33 wk	16.32	16.35	16.73	16.86	0.380	0.117	0.852
34–37 wk	16.49	16.55	16.73	16.82	0.343	0.288	0.955
Cumulative (26–37 wk)	16.37	16.40	16.77	16.84	0.249	0.037	0.905
Albumin weight, g							
26–29 wk	37.64	37.31	37.14	37.31	0.653	0.588	0.595
30–33 wk	38.06	37.91	38.26	38.66	0.551	0.234	0.486
34–37 wk	39.07	38.73	39.30	38.79	0.509	0.111	0.262
Cumulative (26–37 wk)	38.26	37.98	38.23	38.59	0.412	0.354	0.295
Yolk percentage, %							
26–29 wk	27.63	27.54	28.21	28.14	0.444	0.136	0.979
30–33 wk	27.47	27.31	27.57	27.52	0.561	0.812	0.891
34–37 wk	27.21	27.19	27.12	26.98	0.591	0.688	0.886
Cumulative (26–37 wk)	27.44	27.35	27.63	27.55	0.340	0.570	0.997
Albumin percentage, %							
26–29 wk	63.81	63.00	62.86	62.87	0.770	0.052	0.459
30–33 wk	64.04	63.33	63.05	63.10	0.692	0.177	0.451
34–37 wk	64.47	63.60	63.67	63.81	0.542	0.285	0.209
Cumulative (26–37 wk)	64.11	63.31	62.99	63.06	0.512	0.049	0.252

SEM, standard error of means.

1 CON, drinking water without wild ginseng; WGD1, 0.05% wild ginseng in drinking water; WGD2, 0.1% wild ginseng in drinking water; WGD3, 0.5% wild ginseng in drinking water. The same letter superscript indicates no significant difference, whereas different letter superscript indicates significant differences (*P* < 0.05).

### Fecal Microbiota

The effect of supplementing WG in drinking water on the fecal microbiota of laying hen is shown in Table 6. There was no significant difference in wk 26 to 29 for *Lactobacillus*. However, *Lactobacillus* was linearly higher (*p* < 0.05) from wk 30 to 37 at WDG3 compared with CON. There was no significant difference in *Bifidobacterium* and *Clostridium* spp*.* across all treatments and wk. However, *E. coli* was linearly lower in the WGD2 and WGD3 compared with CON throughout wk 26 to 37.

**Table 6:** Effects of water supplemented wild ginseng on the fecal microbiota of laying hens.

Items^1^ DNA (ng)	CON	WGD1	WGD2	WGD3	SEM	*Linear*	*Quadratic*
*Lactobacillus* spp*.*							
26–29 wk	1.25	1.26	1.28	1.28	0.044	0.462	0.993
30–33 wk	1.31^b^	1.32^b^	1.38^ab^	1.42^a^	0.028	0.001	0.603
34–37 wk	1.33	1.34^b^	1.40^ab^	1.48^a^	0.041	0.002	0.257
*Bifidobacterium* spp.							
26–29 wk	0.70	0.73	0.76	0.75	0.046	0.498	0.538
30–33 wk	0.74	0.76	0.77	0.78	0.034	0.197	0.639
34–37 wk	0.71	0.74	0.77	0.80	0.044	0.800	0.477
*Clostridium* spp.							
26–29 wk	0.48	0.53	0.44	0.47	0.042	0.302	0.543
30–33 wk	0.55	0.53	0.54	0.50	0.033	0.242	0.758
34–37 wk	0.50	0.53	0.53	0.51	0.042	0.056	0.997
*Escherichia coli* spp.							
26–29 wk	0.40^a^	0.37^ab^	0.35^b^	0.34^b^	0.027	0.048	0.752
30–33 wk	0.42^a^	0.40^a^	0.37^b^	0.34^b^	0.048	0.008	0.600
34–37 wk	0.40^a^	0.40^a^	0.34^b^	0.29^b^	0.028	<0.001	0.242

SEM, standard error of means.

1 CON, drinking water without wild ginseng; WGD1, 0.05% wild ginseng in drinking water; WGD2, 0.1% wild ginseng in drinking water; WGD3, 0.5% wild ginseng in drinking water. The same letter superscript indicates no significant difference, whereas different letter superscript indicates significant differences (*P* < 0.05).

### Immunomodulatory Effect

**Figure 1:**
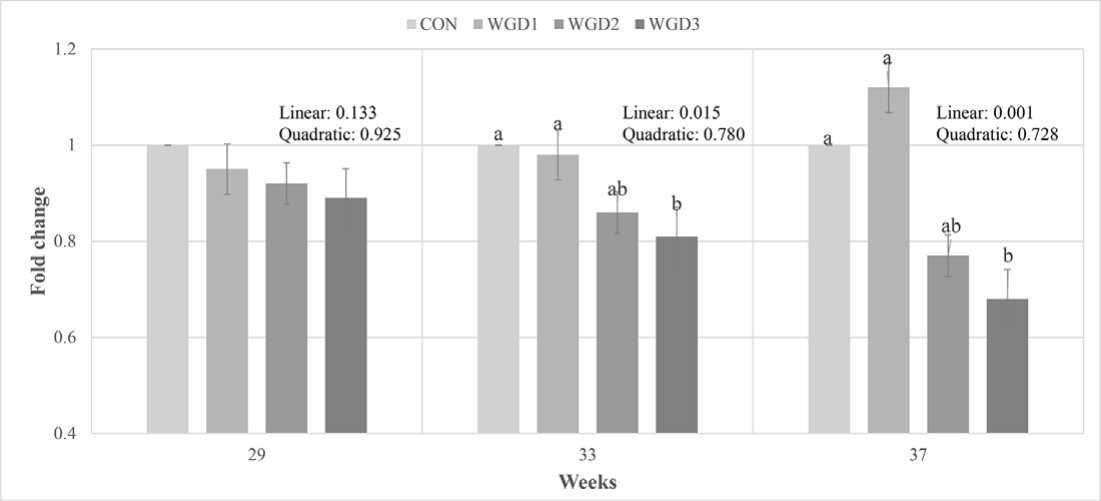
Effects of water supplemented wild ginseng on the relative expression of TNF-α. CON, drinking water without wild ginseng; WGD1, 0.05% wild ginseng in drinking water; WGD2, 0.1% wild ginseng in drinking water; WGD3, 0.5% wild ginseng in drinking water. Data were presented as mean values and standard errors of means. The same letter superscript indicates no significant difference, whereas different letter superscript indicates significant differences (*P* < 0.05).

The effect of supplementing WG in the drinking water of laying hen on the relative expression of TNF-α is shown in Figure 1. There was no significant difference in wk 26 to 29 across all treatments. However, the expression of TNF-α was linearly lower (*p* < 0.05) from wk 33 to 37 in WGD3 compared with CON and WGD1.

### Yolk Ginseng Saponin / Ginsenoside Content

**Figure 2:**
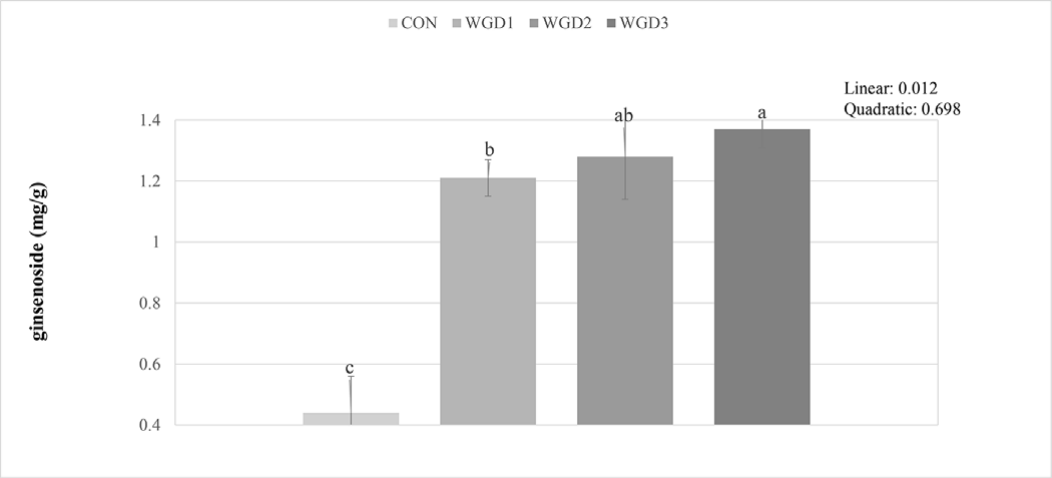
Effects of water supplemented wild ginseng on the ginsenoside/ginseng saponin content in yolk. CON, drinking water without wild ginseng; WGD1, 0.05% wild ginseng in drinking water; WGD2, 0.1% wild ginseng in drinking water; WGD3, 0.5% wild ginseng in drinking water. Data were presented as mean values and standard errors of means. The same letter superscript indicates no significant difference, whereas different letter superscript indicates significant differences (*P* < 0.05).

Figure 2 shows the effects of supplementing WG in drinking water of laying hen on the GS content in egg yolk. The GS content of the egg yolk was linearly higher (*p* < 0.05) in the WGD3 compared with CON.

## DISCUSSION

The supplementation of WG in drinking water in our study had no significant effect on ADFI and BW but improved FCR. This is in contrast with the suggestion by Jenkins and Atwal (1994); Kang et al. (2016) which stated that dietary saponins, the primary bioactive substance in ginseng had a negative effect on growth performance. The improved FCR in our study may be attributed to the potency of herbs and their extracts as a potential modulator of the digestive enzyme activities in the stomach. The contrary opinion in our experiment may be due to the medium of WG supplementation.

The inclusion of WG at WGD3 in the peak phase of our study also improved HDEP, AEW, EM, fecal microbiota quality, and TNF-α which could be kindred to the prevalence of GS in the yolk. GS are the principal constituents of ginseng and its by-product, with a beneficial effect on the reproductive tract and other tissues (Hamid et al., 2020; Jung et al., 2023). Our experiment identified the direct relationship between microbiota quality, TNF-α, laying performance, GS in egg yolk.

Laying hens are prone to secreting higher reactive oxygen species resulting in intestinal oxidative stress and tumor necrosis due to their genetic preference for long-laying capacity (Jian et al., 2021; Sharma et al., 2024), especially during the peak period between 32-36 wk with the most pathogenic susceptibility changes as infection can be attributed to sex hormone. A report by Fang et al. (2021); Rychlik et al. (2023) explained that *E. coli* colonization in the oviduct was higher as estrogen levels increased at the peak stage. This unravels that high egg production may weaken the immune system of hens, making them susceptible to infection by stimulating a wide distribution of *E. coli* in excretes responsible for the popularity of colibacillosis in poultry. In our study, WG reduced the harmful effects of *E. coli* by elevating the level of *Lactobacillus* spp. leading to a reduction in the synthesis of inflammatory cytokine at the peak laying stage of our experiment. *Lactobacillus* is a growth-promoting bacteria with the ability to ameliorate laying performance and FCR via the manipulation of intestinal microbiota content to improve the survivability of non-harmful co-anaerobic and gram-positive microbiota, inhibit the proliferation of pathogenic microbiota, and improve nutrient utilization and absorption (Xu et al., 2023). This is in agreement with the study of Liu et al. (2018), Zhang et al. (2023). In their study, it was proven that the supplementation of ginseng increased the level of *Lactobacillus* and *Bifidobacterium* and reduced pathogenic *Colibacilus and Peptostreptococcaceae* which can result in inflammation.

An important relationship between inflammatory TNF-α and egg production worthy of mention is apoptosis. TNF-α is an inducer of dead cells in the ovarian tissues of the granulosa cells of laying hen which accelerates a rapid reduction in oocyte and follicle numbers (Morrison and Marcinkiewicz, 2002). Therefore, the improved laying performance in the WGD3 can also be attributed to the effect of GS in mitigating the activities of TNF-α, thereby alleviating the granular cells known to be an essential part of the ovarian follicle responsible for egg formation in layer hen.

## CONCLUSIONS

In summary, the supplementation of WG in water, as evidenced by its presence in egg yolk can be surmised to have contributed to the increased fecal *Lactobacillus*, and reduced level of *E. coli,* thereby mitigating the adverse effect of TNF-α and promoting the FCR, HDEP, EM and AEW at WGD3 with 0.5% WG in drinking water of laying hen from 30-37 wk.
